# Functional outcome after recurrent patellar dislocation: Comparison of two surgical techniques—Medial patellofemoral ligament reconstruction (MPFL) vs. Elmslie Trillat procedure

**DOI:** 10.1007/s00508-019-01570-3

**Published:** 2019-11-11

**Authors:** Mohammad Keilani, Stefano Palma, Richard Crevenna, Camilla Gaudart, Timothy Hasenöhrl, Martin Reschl, Nadine Huto, Stefan Hajdu, Harald K. Widhalm

**Affiliations:** 1 Departmentof Physical Medicine, Rehabilitation and Occupational Medicine, Medical University of Vienna, Waehringer Guertel 18–20, 1090 Vienna, Austria; 2 Department of Orthopedics and Traumatology, Clinical Division of Traumatology, Medical University of Vienna, Vienna, Austria

**Keywords:** Patellar displacement, Surgery, Muscle strength, Isokinetic testing, Body composition

## Abstract

**Background:**

There is no final consensus regarding the ideal surgical technique for the treatment of patellar dislocation. The aim of this retrospective pilot study was to describe muscle strength, body composition, self-reported physical performance, and pain in male patients after patellar dislocation treatment with two different surgical techniques: medial patellofemoral ligament (MPFL) reconstruction vs. the Elmslie-Trillat procedure.

**Methods:**

Isokinetic testing of knee extensor muscles was performed using a Biodex System 3 pro dynamometer at an angular velocity of 60°/s. Body composition was measured with bioelectrical impedance analysis (Nutribox). Self-reported physical performance and pain were assessed by the SF-36 subscales of physical functioning, role physical and bodily pain. The outcome variables of peak torque normalized to participant’s body mass (Nm/kg), lean body mass, phase angle, self-reported physical performance, and pain were compared between the study groups.

**Results:**

Of the 12 included male patients, 6 had been treated with MPFL reconstruction (age: median = 33 years, range = 18–38 years; BMI: median = 26 kg/m^2^, range = 23–29) and 6 with the Elmslie-Trillat procedure (age: median = 26 years, range = 19–32 years; BMI: median = 23 kg/m^2^, range = 19–28). No statistically significant differences were found between the groups in any outcome parameter of muscle strength, body composition, self-reported physical performance, or pain.

**Conclusions:**

The results of the present pilot study revealed that MPFL reconstruction shows equal results to the Elmslie-Trillat procedure, with respect to isokinetic knee muscle strength, body composition, self-reported physical performance and pain in male patients suffering from recurrent patellar dislocation.

## Introduction

Patellar dislocation is a condition which occurs especially among physically active persons [1]. It has been reported as accounting for 2–3% of all acute knee injuries [1]. The surgical procedures for the treatment of patellar dislocation alter the damaged patella mechanics by the use of several approaches; for example, the relief of tight lateral structures, tensioning of loose medial ligaments and distal realignment of the extensor mechanism [2].

In a recent epidemiological study, medial patellofemoral ligament (MPFL) reconstruction was performed in 75% of all patella-stabilizing surgeries and was performed on almost 10% of patients with patellar dislocation [3]. The MPFL reconstruction is intended to allow the patient a faster return to normal activity [4]. Further advantages of MPFL reconstruction are the low recurrence rates and the restoration of anatomic structures; however, this is an invasive and technically demanding procedure, requiring an additional graft harvest, and relies on sufficient surgical experience. Furthermore, expensive equipment is required during MPFL reconstruction [4]; however, there is no consensus regarding the ideal surgical technique for the treatment of patellar dislocation [5]. For rehabilitation issues, quadriceps muscle strength seems to be an important factor for a good outcome after knee surgery. Current literature describes deficits in both short-term and long-term isokinetic knee extension strength following patellar dislocation [6, 7]. Notably, to the best of our knowledge, no recent clinical study has compared muscle strength and body composition in patients after treatment with the different surgical techniques. Therefore, the purpose of this retrospective pilot study was to compare muscle strength, body composition, self-reported physical performance, and pain in patients following treatment for patellar dislocation with either surgical technique (MPFL reconstruction vs. the Elmslie-Trillat procedure). It was hypothesized that both surgical techniques would show similar results.

## Material and methods

This study was approved by the ethics committee of the Medical University of Vienna (EK Nr: 1161/2016). The study was performed in accordance with the Declaration of Helsinki (1964). Male patients who had been operated on due to recurrent patella dislocation between 2011 and 2016 with either MPFL reconstruction (MPFL group = group 1) or the Elmslie-Trillat procedure (group 2) were included in the study. Inclusion criteria were recurrent patellar dislocation, surgery using either MPFL reconstruction or the Elmslie-Trillat procedure, stable knee joint, and intact patella. Exclusion criteria were first occurrence of patellar dislocation, patellar fracture, unstable knee joint, age >50 years and body mass index >35 kg/m^2^. All participants were contacted by telephone or by mail and were invited to participate in the follow-up examination. All participants gave informed consent prior to inclusion in the study. The participants were examined at a mean follow-up of 47 months (MPFL group) and 43 months (Elmslie-Trillat procedure group).

### Surgical techniques

#### Medial patellofemoral ligament reconstruction (MPFL)

Performing a MPFL reconstruction [8] requires extraction of a tendon. This is usually taken as a hamstring tendon of the patient’s own body or an allograft is used. The tendon must have a diameter of at least 5 mm and a length of approximately 20 cm, and the ends must be reinforced. An approximately 3‑cm long skin incision is made and prepared on the medial patellar surface, so that the bony medial edge of the patella is well displayed. Thereafter, two wires are drilled parallel in the horizontal plane in the area of the patella and are overdrilled by the use of a cannulated drill. The two suture anchors are connected to the ends of the tendons and fixation of the graft is performed in the bony tunnels by interference screw fixation. Thereafter, the preparation is carried out between two layers in the direction of the flexor side as well as the shuttling of the U‑shaped tendon complex. After this step the isometric point on the distal femur [9], localized 1.3 mm anterior to the posterior cortex and 2.5 mm distal to a perpendicular line intersecting the posterior medial condyle, has to be identified followed by femoral tunnel drilling and fixation of the shuttled tendon by an interference screw in a 20° extension of the knee joint.

#### Elmslie-Trillat procedure

In contrast to pure soft tissue surgery, as with the MPFL plastic procedure the Elmslie-Trillat procedure [10] is an osteotomy procedure. After a long incision of the skin from the tip of the patella to just below the tibial tuberosity, parapatellar preparation is performed on both sides. If the tuberosity has been shown to be clean, it is undercut at a distance of about 4 cm with an oscillating saw from the lateral side. After the knee joint is moved through it, the osteotomized piece of bone must be carefully shifted in the medial direction. Following a good position being achieved, two drill wires are placed in the bone piece, and the displaced tuberosity fragment is fixed with two large fragment cortical screws.

### Follow-up examination

Follow-up consultations included obtaining a medical history as well as assessment of self-reported physical performance and pain, physical examinations, isokinetic quadriceps strength measurements and body composition assessments.

### Assessment of muscular strength

In the present study, isokinetic testing of the thigh of the operated knee (isokinetic knee extension) was performed using a Biodex System 3 Pro dynamometer (Biodex Medical Systems, Shirley, NY, USA) [11]. Isokinetic testing is the gold standard for the objective measurement of muscle strength in vivo, and was therefore used for the assessment of thigh muscle strength in previous clinical studies [12–14]. The testing was performed at an angular velocity of 60°/s. Isokinetic testing at 60°/s is suggested by the manufacturer for the evaluation of maximum voluntary strength, and has been shown to be valuable and valid for measuring maximum muscular strength [11, 12]. The device was calibrated for torque and range of motion. The measurements were performed according to the manufacturer’s description. Test protocols were established based on the Biodex System 3 Pro manual [11]. Prior to isokinetic testing, a 5-min warm up was performed using an exercise bicycle (ergometer). The participants were then stabilized in the test chair with abdominal and shoulder belts. The anatomical axis of rotation was matched to the dynamometer axis through manual palpation and visual examination. For the purpose of acclimatization, testing was explained to the participants, and they were then asked to perform submaximal isokinetic contractions, in order to feel confident about the testing [11]. A total of five repetitions of isokinetic testing were performed at an angular speed of 60°/s for knee extension and knee flexion [8]. Muscle strength was assessed as the moment of maximum torque during knee movement at a constant isokinetic velocity of 60°/s (PT = peak torque normalized to participant’s body weight in Nm/kg). The highest achieved value was used [11, 14]. In order to reduce interrater variability, all measurements were conducted by the same investigator, who gave standardized verbal encouragement during the procedure [11]. To assess the strength of the skeletal muscle in the patients, the maximum PT of the thigh muscles (knee extensors) was calculated for comparison between the two study groups. Furthermore, a difference compared to age and sex-predicated expected values was calculated [11, 14].

### Assessment of body composition

Body composition was measured using bioelectrical impedance analysis (BIA), which represents the measurement of electrical resistance in an organic body [http://www.data-input.de/media/pdf_english_2014/instructions-for-use-nutribox], in which a field of alternating electric current is produced in the patient’s body (via electrodes on the skin). The measurements are made between electrodes placed manually on the wrist and ankle. In the present study, the measurements were conducted according to the manufacturer’s operating manual [15]. The BIA was performed using the impedance analysis apparatus data input nutribox rev. 1.0. Body composition was assessed by lean body mass (kg) and phase angle, which is proportional to the cell mass (ratio of body cell mass to fat-free mass). The assessment of body composition was compared between the two study groups.

### Assessment of self-reported physical performance and pain

Self-reported physical performance and pain was assessed using the German version of the SF-36 health survey [16]. The SF-36 health survey is a well-accepted generic patient-related outcome measure for assessing eight different domains of health-related quality of life (HRQOL) [16]. In this study, patients filled in the subscales entitled physical functioning, role physical and bodily pain of the German version of the SF-36 health survey [16]. For each of the three scales, the responses to the questions are summarized and then converted to a scale of 0–100 points [16]. The main advantage of the SF-36 health survey is that the test is applicable to many disease groups as well as the general population, so that it can be used to compare ill patients with healthy populations [16]. This allows the exploration of the effects of specific diseases on HRQOL and compare them the impact of aging and associated ailments. It has been shown that the use of a generic HRQOL questionnaire can help physicians to look beyond “what is wrong” with their patients [16]. The descriptions of self-reported physical performance and pain values for the SF-36 subscales of physical functioning, role physical and bodily pain were used for the comparison between study groups [16].

### Statistical analysis

Descriptive statistics were calculated. Due to the small sample size, the Mann-Whitney U-test for two independent samples was performed to compare the correlations between peak torque, lean body mass, body fat (%) and the SF-36 subscales of physical functioning, role physical and bodily pain.* P* values ≤0.05 were considered to be statistically significant. No correction for multiple testing was calculated, due to the small sample size. Statistical analyses were performed using IBM SPSS v25.

## Results

Of the 14 patients who met the inclusion criteria, 12 were available for the follow-up assessment. Of those 12 patients, 6 had been treated with MPFL reconstruction and 6 with the Elmslie-Trillat procedure. The available demographic data and outcome variables of the study population (*n* = 12) are presented in Table 1.

**Table 1:** Descriptive presentation of demographic data and the outcome variables (muscle strength, body composition, self-reported physical performance and pain) of the study population (*n* = 12)

Variable	Group 1 (median, range)	Group 2 (median, range)
Age (years)	33, 18–38	26, 19–32
*N*	6	6
BMI (kg/m^2^)	26, 23–29	23, 19–28
PT (Nm/kg)	205, 179–275	210, 195–297
Lean body mass (kg)	67, 61–71	63, 55–74
Phase angle (°)	6.7, 6.1–7.8	6.7, 6.0–7.9
Physical functioning	100, 83–100	94, 89–100
Role physical	100, 75–100	100, 75–100
Bodily pain	90, 58–100	100, 80–100

*Group 1* medial patellofemoral ligament reconstruction, *group 2* Elmslie-Trillat procedure, *BMI* body mass index, *PT* peak torque at 60°/s of knee extensors/normalized to participant’s body weight, (Nm/kg)

The Mann-Whitney U-test showed no significant differences in the outcome variables between patients who were treated with MPFL reconstruction or the Elmslie-Trillat procedure (Table 2).

**Table 2:** Comparison of outcome parameters between patients after medial patellofemoral ligament reconstruction and the Elmslie-Trillat procedure

Mann-Whitney U-test	*P*-value
Peak torque (Nm/kg)	0.485
Lean body mass (kg)	0.818
Phase angle (°)	0.699
Physical functioning	0.818
Role physical	0.699
Bodily pain	0.116

Peak torque: peak torque at 60°/s of knee extensors/normalized to participant’s body weight (Nm/kg)

During the 60°/s extension, five out of six patients in group 1 (median: 17%) and four out of six patients of group 2 (median: 15%) demonstrated a deficit of PT compared to sex and age-related reference values. Patients of group 1 reported 10% more pain and 6% lower muscle strength compared to patients of group 2 (Table 1); however, these differences were not significant (Table 2).

## Discussion

The results of the present study showed no significant differences in muscle strength, body composition, self-reported physical performance, and pain between the investigated surgical procedures, underlining the relevance of the long-term outcomes of both techniques. In comparison, only a few studies reporting isokinetic muscle strength testing results following surgery for patellar dislocation are known. Some studies have dealt with isokinetic testing after MPFL reconstruction [7, 17, 18]. In a study by Ronga et al. [7], isokinetic muscle strength of the lower extremity after isolated MPFL reconstruction was shown to be weaker than that of the contralateral limb 3.1 years postoperatively. These results are similar to those of another isokinetic cohort study, where isokinetic measurements revealed a decline in muscle strength compared to the contralateral limb at 6 months after surgery [17]. The results of a recent study showed that knee extensor strength improved after MPFL reconstruction, although an approximately 20% deficit against the non-operated leg remained even 2 years after the operation [18]. The findings of the present study appear to be in accordance with the recent literature concerning long-term outcomes after MPFL reconstruction. The lack of a final consensus regarding the best surgical technique following patellar dislocation prompted assessment of muscle strength after the different surgical techniques (MPFL and the Elmslie-Trillat procedure). In a systematic review and meta-analysis by Lee et al. [4], functional recovery for patellar dislocation post-MPFL reconstruction vs. medial soft tissue realignment surgery was investigated by assessing different clinical scores, such as the Kujala score, the Lysholm score, the Tegner score and pain scores. The authors concluded that MPFL reconstruction results in more favorable clinical outcomes than medial soft tissue realignment surgery in patients with recurrent patellar dislocation [4]; however, to the best of our knowledge, no clinical study has compared the different surgical techniques in term of knee muscle strength and body composition. A biomechanical study by Mountney et al. [19] revealed that tensile strength seemed to be greater after MPFL reconstruction than sutures alone, and bone anchors with suture anchor reconstruction seemed to be weaker than MPFL and weaker than through-tunnel reconstructions. No significant differences in strength were found between the MPFL and through-tunnel procedures [19].

Quadriceps muscle strength is acknowledged to relate to different basic activities of daily living (walking, stair climbing), and to falling [12]. Body composition is associated with physical performance in individuals with other knee disorders such as osteoarthritis [20]. Quadriceps weakness seems to predict declining knee joint function because the quadriceps muscle is known to be a primary dynamic contributing factor to knee stability. This can lead to pathological loading and subsequent structural damage, including to the bone marrow as well as cartilage and bone lesions [21]. Such structural damage can cause chronic knee pain, which leads to a vicious circle, consisting of decreased physical activity causing muscle weakness leading to a further decline in activity. This is especially true in morbidly obese patients at high risk of suffering from patellar dislocation [22]. Muscle weakness, the related loss of muscle mass, and pathological alterations in body composition can contribute in the long term to metabolic and cardiovascular diseases [23]. Fisher et al. [24, 25] reported in a systematic review on MPFL reconstruction and rehabilitation that quadriceps dysfunction was the most frequent complication after surgery. Quadriceps muscle weakness was found in both study groups compared to age and sex-dependant predicted values; however, this does not indicate which surgical technique should be performed for patellar dislocation. Different factors such as quadriceps angle and tibial tubercle-trochlear groove distance are important for the decision made by the surgeon. Preoperative diagnostic imaging and arthroscopy are used to evaluate the patellofemoral articular surfaces, looking for any chondral damage.

The advantages and disadvantages of the different surgical techniques after recurrent patellar dislocation have been discussed in the literature. With repect to postoperative rehabilitation, during the first 4–6 weeks after MPFL reconstruction, partial weight-bearing with the use of crutches and a brace is usually allowed until an independent straight leg raise can be performed [24]. In contrast, after the Elmslie-Trillat procedure no weight-bearing is allowed for 4–6 weeks postsurgery, followed by a limited increase in load and a limited flexion of 60° after a radiological examination showing healing of the medialized bone block [26]. Therefore, MPFL reconstruction is supposed to allow the patient to recover muscle strength faster. The results showed comparable muscle strength between the investigated surgical techniques suggesting this difference equalizes in the long term.

In the present study, the testing was performed at an angular velocity of 60°/s, because this is suggested by the manufacturer for the evaluation of maximum voluntary strength, and has also been recently shown to be useful and valid for assessing maximum muscular strength [11, 27]. Furthermore, this study assessed isokinetic strength and related it to the weight of the patient (Nm/kg), because strength in most muscle groups has been proven to be related not only to age and height, but also to body mass [14]. The time from surgery to follow-up was 47 months (MPFL group) and 43 months (Elmslie-Trillat procedure group), representing a long-term assessment.

## Limitations

The reason for these differences remains unclear, due to the lack of data prior to surgery. The retrospective study design and the small sample size are the main limitations of this study. The sample size was too low to enable strong conclusions to be drawn, due to the fact that the number of patients treated surgically is generally low [1, 3]. Nevertheless, this was primarily a very small pilot study innovatively comparing muscle strength, body composition and functional outcome between MPFL reconstruction and the Elmslie-Trillat procedure. Therefore, the results of this study should be interpreted with caution. A further limitation was that body composition was not analyzed using gold standard measurement methods, such as dual-energy x‑ray absorptiometry or air displacement plethysmography. Moreover, BIA works sufficiently well in healthy people and in patients with stable water and electrolyte balance. None of the participants showed pathological hydration or were at the extremes of the body mass index ranges. Furthermore, the age discrepancy between group 1 and 2 (33 years vs 26 years, respectively) may represent a systematic bias. This emphasizes that the results do not allow strong conclusions to be drawn; however, muscle strength was also compared to sex and age-related reference values, and the results revealed no notable difference between the two study groups.

## Conclusion

The results of the present pilot study revealed that MPFL reconstruction shows equal results to the Elmslie-Trillat procedure, with respect to isokinetic knee muscle strength, body composition, self-reported physical performance and pain in male patients suffering from traumatic recurrent patellar dislocation.
